# Reporting of Human Genome Epidemiology (HuGE) association studies: An empirical assessment

**DOI:** 10.1186/1471-2288-8-31

**Published:** 2008-05-20

**Authors:** Ajay Yesupriya, Evangelos Evangelou, Fotini K Kavvoura, Nikolaos A Patsopoulos, Melinda Clyne, Matthew C Walsh, Bruce K Lin, Wei Yu, Marta Gwinn, John PA Ioannidis, Muin J Khoury

**Affiliations:** 1 National Office of Public Health Genomics, Coordinating Center for Health Promotion, Centers for Disease Control and Prevention, Atlanta, USA; 2 Clinical and Molecular Epidemiology Unit, Department of Hygiene and Epidemiology, University of Ioannina, School of Medicine, Ioannina, Greece; 3 Department of Population Health Sciences, University of Wisconsin, Madison, USA; 4 Office of the Medical Director, March of Dimes Birth Defects Foundation, White Plains, USA

## Abstract

**Background:**

Several thousand human genome epidemiology association studies are published every year investigating the relationship between common genetic variants and diverse phenotypes. Transparent reporting of study methods and results allows readers to better assess the validity of study findings. Here, we document reporting practices of human genome epidemiology studies.

**Methods:**

Articles were randomly selected from a continuously updated database of human genome epidemiology association studies to be representative of genetic epidemiology literature. The main analysis evaluated 315 articles published in 2001–2003. For a comparative update, we evaluated 28 more recent articles published in 2006, focusing on issues that were poorly reported in 2001–2003.

**Results:**

During both time periods, most studies comprised relatively small study populations and examined one or more genetic variants within a single gene. Articles were inconsistent in reporting the data needed to assess selection bias and the methods used to minimize misclassification (of the genotype, outcome, and environmental exposure) or to identify population stratification. Statistical power, the use of unrelated study participants, and the use of replicate samples were reported more often in articles published during 2006 when compared with the earlier sample.

**Conclusion:**

We conclude that many items needed to assess error and bias in human genome epidemiology association studies are not consistently reported. Although some improvements were seen over time, reporting guidelines and online supplemental material may help enhance the transparency of this literature.

## Background

Human genome epidemiology (HuGE) is a rapidly emerging scientific field that examines the influence of genomic variation on human health [1-4]. Although a large and rapidly increasing number of studies have investigated the associations between genetic variants and the risks of common diseases through observational epidemiology, few significant associations have been shown to be reproducible in multiple studies [5,6]. Transparent reporting of the study populations, methods of data collection, analytic methods, and study inferences may help readers better identify issues that can affect the reproducibility of genetic association studies. Here, we conduct a detailed evaluation of reporting practices for HuGE association studies.

## Methods

In 2001, the Human Genome Epidemiology Network (HuGENet) established the HuGE Published Literature database (HuGE Pub Lit), a continually updated, searchable, online database of population-based, genetic epidemiology articles [7]. Relevant studies are identified weekly from NCBI PubMed [8] by a genetic epidemiologist who records the study design, genes and diseases of interest, and interacting environmental factors [7]. As of May 21, 2007, this database included a total of 27,386 articles that examined genotype-phenotype associations (both qualitative and quantitative traits) published in 2,773 journals. Further details regarding the contents of this database have been previously described [7]. This information along with the title, contributing authors, abstract, journal, date of publication, and the unique PubMed Identifier (PMID) are deposited in the HuGE Pub Lit database [7]. To select articles for this analysis, we queried the HuGE Pub Lit database for population-based studies that used observational study designs (i.e., case-control, cohort, and cross-sectional studies) to investigate gene-disease associations, interactions between genetic variants (interlocus or gene-gene interactions), or gene-environment interactions. Family-based linkage studies were not collected systematically in HuGE Pub Lit and, therefore, were not included in this study. In addition, we restricted our analysis to full text articles because studies presented only as concise summaries (e.g., as letters or abstracts) could have increased the heterogeneity of our sample.

Our evaluation was designed in 2004 and data collection and analyses were conducted in 2004–2007. For the main analysis, we drew a five percent simple random sample (SRS) of articles that were returned by the query described above, published from 2001 to 2003, and curated in HuGE Pub Lit before May 30th, 2004 (n = 8,115) to yield a dataset of 406 articles. To provide an updated description of reporting practices and to assess improvements in reporting, we randomly selected (SRS) 40 articles that were published during 2006 from articles that were returned by our database query, added to PubMed in 2006, and curated in HuGE Pub Lit before May 18, 2007 (n = 5,353). After each article was read, 91 from 2001–2003 and 12 from 2006 were excluded from the analysis for the following reasons: not written in English (2001–2003: n = 28, 2006: n = 6), population screening studies (2001–2003: n = 23, 2006: n = 0), clinical trials or pharmacogenomic studies (2001–2003: n = 16, 2006: n = 3), not full-length articles (i.e., letter or abstract) (2001–2003: n = 11, 2006: n = 0), failed to fulfill the inclusion criteria for HuGE Pub Lit [7] on closer scrutiny (2001–2003: n = 6, 2006: n = 1), family studies (2001–2003: n = 3, 2006: n = 2), studies of genetic tests (2001–2003: n = 2, 2006: n = 0), or meta-analyses (2001–2003: n = 2, 2006: n = 0).

Data were abstracted from each original publication in duplicate by two independent data extractors. All discrepancies between the independent extractors were discussed and a consensus was reached.

For the 2001–2003 articles, a standardized abstraction form was developed and piloted for 10 articles; the form was revised according to the results of this pilot study to ensure that the definitions for the collected items were clear and unambiguous. Items on this final form were designed to collect information on the reporting of study design, genotyping method, population stratification, analytical methods (including the analysis of multiple genetic variants and gene-environment interactions), and study inferences. In addition, the final form accommodated different observational study designs, multiple groups of study participants, and the consideration of more than one postulated genetic risk factor as well as additional environmental factors. Articles were coded as potentially misclassifying the disease or environmental exposure status of study participants when the article did not explicitly state that these factors were directly measured for all participants in the study population. When multiple groups of study participants were reported in a study, we recorded the sample size of the largest group for cohort and cross-sectional studies and the largest case and control groups for case-control studies. Items were collected separately for case and control groups for case-control studies and for all study participants regardless of disease status for cohort and cross-sectional studies. For the purpose of this analysis, data collected for case and control groups were combined so that statistics could be calculated for all study participants. Information (e.g., mean or median age and sex distribution) was considered as given for all study participants only if it was provided for all case and control groups. Additionally, for case-control studies, we recorded whether cases and controls were described as drawn from the same population according to one or more of the following definitions: 1) geographic region, 2) clinical population, 3) general population (i.e., ethnic group), or whether information on the choice of suitable controls was missing or incomplete.

Fourteen items were assessed in the HuGE articles published in 2006. These included the number of study participants, genes, polymorphisms, and environmental factors assessed in gene-environment interactions. In addition, we selected ten items that were applicable to all study designs and that had been reported in fewer than 50% of the articles published in 2001–2003.

The data analysis was conducted using SAS 9.13 (SAS Institute, Cary, NC). Counts and percents were calculated for the items abstracted from the articles. Comparisons of articles published in 2006 vs. those published in 2001–2003 used the Mann-Whitney U test for continuous variables and Fisher's exact test for binary variables.

## Results

### HuGE articles published from 2001 to 2003

The 315 articles selected for analysis were published in 194 journals and reported on the findings of 227 case-control, 32 cohort, and 56 cross-sectional studies. In addition to population-based studies, three articles also described family-based analyses. Data pertaining exclusively to these family-based analyses were not included in this report. As shown in Table 1, most articles (75.9 percent) reported sample sizes of fewer than 500 study participants; 9.2 percent reported sample sizes greater than or equal to 1,000 (median = 265, interquartile range (IQR) 142–471). Statistical power was reported in 12.7 percent of articles. Multiple study populations (e.g., more than one case or control group) were reported in 25.4 percent of the articles. Most of the studies provided at least some information about the origin (87.9 percent) and the enrollment criteria (97.5 percent) of the study participants. The sex distribution was provided in three-quarters of the articles, whereas the median or mean age of the study participants and a measure of the variation around this value (e.g., IQR or standard deviation) were reported for 65.4 percent and 54.6 percent, respectively. One in six articles explicitly stated that the study participants were unrelated. We estimated that 11.8 percent of studies could have misclassified the outcome of interest.

**Table 1:** Reporting characteristics of the study design for 315 randomly selected HuGE articles (2001–2003)

**Reporting characteristic**	**Count**	**Percent**
Number of study participants		
< 100	49	15.6
100–499	190	60.3
500–999	47	14.9
>= 1000	29	9.2
Reported the available power of the study		
No	275	87.3
Yes	40	12.7
Reported that multiple study populations or case or control groups were used		
No	235	74.6
Yes	80	25.4
Provided any information on the origin of the study participants		
No	38	12.1
Yes	277	87.9
Provided any information on the enrollment criteria for the study participants		
No	8	2.5
Yes	307	97.5
Sex distribution reported for all study participants		
No	84	26.7
Yes	231	73.3
Mean or median age reported for all study participants		
No	109	34.6
Yes	206	65.4
Standard deviation or interquartile range reported for all study participants		
No	143	45.4
Yes	172	54.6
Explicitly stated the use of unrelated study participants		
No	259	82.2
Yes	56	17.8
Potential for outcome misclassification		
No	272	86.3
Unclear	6	1.9
Yes	37	11.8

Seven percent of studies reported that the genotyping results were validated with the use of replicate samples, and an additional 9.8 percent reported that a different method of validation was used (Table 2). A blind evaluation of the genetic test to the outcome (11.1 percent) or of the outcome to the genetic test (3.8 percent) was rarely reported. Few articles reported that any potential participants had been excluded (11.8 percent) or commented on the number of samples that could not be genotyped (15.6 percent).

**Table 2:** Reporting characteristics of the genotyping method for 315 randomly selected HuGE articles (2001–2003)

**Reporting characteristic**	**Count**	**Percent**
Reported that the genotyping results were validated by using duplicate samples		
No	293	93.0
Yes	22	7.0
Reported that the genotyping results were validated by using a different method		
No	284	90.2
Yes	31	9.8
Reported that the evaluation of the genetic test was blind to the outcomes or phenotypes		
Blind	35	11.1
Unclear	280	88.9
Reported that the evaluation of the outcomes or phenotypes was blind to the genetic test		
Blind	12	3.8
Unclear	303	96.2
Reported that individuals were excluded from the original group(s) of study participants		
No	278	88.2
Yes	37	11.8
Reported that several samples could not be genotyped		
No	266	84.4
Yes	49	15.6

As shown in Table 3, almost 60 percent of the articles indicated that all study participants were drawn from the same ethnic population, whereas 9.5 percent reported that the study population included more than one ethnic group. Most of these articles (76.7 percent) either stratified by or controlled for ethnicity; however, a few (23.3 percent) pooled ethnic groups together or did not provide clear information on how data from different ethnic groups were analyzed. The use of unlinked genetic markers to assess population stratification was extremely rare (0.6 percent). Among case-control studies (n = 227), two-thirds indicated that the cases and controls were drawn from the same geographic area; one in five indicated that cases and controls were drawn from the same clinical population, and one-quarter indicated that cases and controls were drawn from the same general population. More than one-third of these articles were unclear about the source populations or reported no information at all on this aspect.

**Table 3:** Reporting characteristics of population stratification for 315 randomly selected HuGE articles (2001–2003)

**Reporting characteristic**	**Count**	**Percent**
Explicitly stated that all study participants were drawn from the same ethnic population		
Unclear	130	41.3
Stated	185	58.7
Analysis conducted by using different ethnic groups		
No	285	90.5
Yes	30	9.5
If different ethnic groups were included, how was ethnicity treated in the analysis (*n *= 30)		
Stratified by or adjusted for ethnic groups	23	76.7
Pooled ethnic groups together	2	6.7
Unclear	5	16.6
Reported that unlinked genetic markers were used to identify population stratification		
No	313	99.4
Yes	2	0.6
Reported that cases and controls were drawn from the same population in regards to geography (*n *= 227)		
No	79	34.8
Yes	148	65.2
Reported that cases and controls were drawn from the same population in regards to the clinical population (*n *= 227)		
No	180	79.3
Yes	47	20.7
Reported that cases and controls were drawn from the same population in regards to the general population (*n *= 227)		
No	167	73.6
Yes	60	26.4
Reported unclear or no information regarding the population from which cases and controls were drawn (*n *= 227)		
No	142	62.6
Yes	85	37.4

Approximately one-half of the articles stated that they examined whether the study populations were in Hardy-Weinberg equilibrium; of these, 6.6 percent reported that the genotype frequencies deviated from those expected under Hardy-Weinberg equilibrium (Table 4). Summary data (e.g., genotype/allele frequencies presented in a tabular format) were reported on all genetic variants of interest for the outcomes in 87 percent of articles. Analysis using alleles (54.6 percent) was less common than analysis using genotypes (85.7 percent). When genotypes were analyzed, a considerable proportion of articles reported on specific genetic comparisons based on dominant or recessive models (20.7 percent); among these studies, 41.1 percent provided a justification for using the selected model. One in ten articles reported corrections for multiple comparisons; most (70.0 percent) used a Bonferroni correction as the method of adjustment. One article reported using both Tukey's and Scheffe's tests to control for multiple comparisons [9].

**Table 4:** Reporting characteristics of the analytic methodology and study inferences for 315 randomly selected HuGE articles (2001–2003)

**Reporting characteristic**	**Count**	**Percent**
Reported that all genetic variants were examined for Hardy-Weinberg equilibrium		
No	164	52.1
Yes	151	47.9
If Hardy-Weinberg equilibrium was reported, did any polymorphism reportedly fail Hardy-Weinberg equilibrium (*n *= 151)		
No	141	93.4
Yes	10	6.6
Summary data reported on all genetic variants of interest for all outcomes		
No	41	13.0
Yes	274	87.0
Reported that analyses were conducted by using allele-based genetic comparisons		
No	143	45.4
Yes	172	54.6
Reported that analyses were conducted by using genotype-based genetic comparisons		
No	45	14.3
Yes	270	85.7
If the analyses were conducted by using genotypes, were selected comparisons or all possible comparisons assessed (*n *= 270)		
All possible	214	79.3
Selected	56	20.7
Justifications given for the selection of specific genetic comparisons (*n *= 56)		
No	33	58.9
Yes	23	41.1
Adjustment for multiple comparisons used		
No	276	87.6
Yes	39	12.4
If an adjustment for multiple comparisons was used, type of adjustment was (*n *= 40, one article used two methods)		
Bonferroni	28	70.0
Fischer's post hoc	1	2.5
Monte Carlo simulations	1	2.5
Scheffe's test	2	5.0
Tukey's test	3	7.5
Unknown	5	12.5
Authors discussed the public health, medical, or clinical implications of their findings		
No	193	61.3
Yes (any mention)	122	38.7
Authors stated that this is the first study on the specific issue		
No	266	84.4
Yes	49	15.6
Clear reference made to the first study on the specific issue (*n *= 266)		
No	243	91.4
Yes	23	8.6
Clear reference made to a systematic review		
No	297	94.3
Yes	18	5.7
Clear reference made to a non-systematic review		
No	309	98.1
Yes	6	1.9

Overall, less than 40 percent of the articles discussed the public health, medical, or clinical implications of their findings. Less than one in six articles claimed to be the first to analyze a particular association. For the articles that did not make this claim, 8.6 percent clearly made reference to the first study on the issue. Six percent of articles clearly referenced a systematic review, and 1.9 percent referenced a non-systematic review.

Nearly two-thirds of the articles (N = 201) investigated multiple genetic variants, often in more than one gene (Table 5). These studies varied in their reporting of linkage disequilibrium (22.9 percent), haplotype analysis (21.4 percent), and gene-gene interactions (24.4 percent). When articles reported on interlocus or gene-gene interactions, slightly over half estimated the relative risk of the phenotypic outcome as an odds ratio; only 4.1 percent reported an absolute difference, and none presented an attributable fraction. The remainder did not present a measure of risk. One-half of the studies that reported on interlocus or gene-gene interactions reported that at least one of these interactions was statistically significant.

**Table 5:** Reporting characteristics of the analysis of multiple genetic variants for 315 randomly selected HuGE articles (2001–2003)

**Reporting characteristic**	**Count**	**Percent**
Number of genes analyzed		
1	188	59.7
2	71	22.5
>= 3	56	17.8
Number of genetic variants analyzed		
1	114	36.2
2	85	27.0
3	56	17.8
4	21	6.6
>= 5	39	12.4
Reported on linkage disequilibrium (among those studying 2 or more polymorphisms) (*n *= 201)		
No	155	77.1
Yes	46	22.9
Reported on an analysis using haplotypes (among those studying 2 or more polymorphisms) (*n *= 201)		
No	158	78.6
Yes	43	21.4
Reported on a interlocus or gene-gene interaction (among those studying 2 or more polymorphisms) (*n *= 201)		
No	152	75.6
Yes	49	24.4
If interlocus or gene-gene interactions were assessed, was risk quantified as an odds ratio or risk ratio (*n *= 49)		
No	22	44.9
Yes	27	55.1
If interlocus or gene-gene interactions were assessed, was risk quantified as an absolute difference (*n *= 49)		
No	47	95.9
Yes	2	4.1
If interlocus or gene-gene interactions were assessed, was risk quantified as an attributable fraction (*n *= 49)		
No	49	100.0
Yes	0	0.0
If interlocus or gene-gene interactions were assessed, claim was made of a statistically significant interaction (*n *= 49)		
No	24	49.0
Yes	25	51.0

Gene-environment interactions were discussed in 15.2 percent (n = 48) of the articles (Table 6). Among these articles, 70.8 percent examined one environmental factor, 20.8 percent examined two, and 8.4 percent examined three or more. We estimated that the potential to misclassify the environmental factor was present in as many as three-fourths of the articles. Very few studies (6.2 percent) presented a description of the possible sources of error in the measurement of the environmental factor or reported the use of dose-dependent models. None of the articles indicated whether the assessment of the environmental factor was blinded to genotype or whether laboratory staff performing the genetic tests was blind to the environmental factor. Risk was quantified as a risk or odds ratio in slightly more than one-half of these studies; however, none reported absolute differences or attributable fractions. A statistically significant gene-environment interaction was reported in 29.2 percent of the papers.

**Table 6:** Reporting characteristics for the analysis of interacting environmental factors for randomly selected HuGE articles (2001–2003)

**Reporting characteristic**	Count*	**Percent**
Number of environmental factors assessed in gene-environment interactions		
1	34	70.8
2	10	20.8
>= 3	4	8.4
Potential for misclassification of the environmental factors		
No	7	14.6
Unclear	4	8.3
Yes	37	77.1
Provided a description of possible error in the measurement of the environmental factor		
No	45	93.8
Yes	3	6.2
Use of dose-depending models		
No	45	93.8
Yes	3	6.2
Reported that the evaluation of the genetic test was blind to the environmental factor		
Blind	0	0.0
Unclear	48	100.0
Reported that the evaluation of the environmental factor was blind to the genetic test		
Blind	0	0.0
Unclear	48	100.0
If gene-environmental interactions were assessed, was risk quantified as an odds ratio or risk ratio		
No	22	45.8
Yes	26	54.2
If gene-environmental interactions were assessed, was risk quantified as an absolute difference		
No	48	100.0
Yes	0	0.0
If gene-environmental interactions were assessed, was risk quantified as an attributable fraction		
No	48	100.0
Yes	0	0.0
Claim of a statistically significant gene-environmental interaction		
No	34	70.8
Yes	14	29.2

* limited to the 48 studies that addressed at least 1 environmental factor

### Comparison of reporting practices in 2001–2003 with 2006

The number of study participants, genes and polymorphisms analyzed, and environmental factors examined in gene-environment interactions were similar for the two time periods (Table 7). Articles in the 2006 sample tended to use sample sizes of less than 500 (75.0 percent) and report on a single gene (75.0 percent), multiple genetic variants (64.3 percent), and no gene-environment interactions (92.9 percent).

**Table 7:** Comparison of the reporting characteristics of HuGE articles from two time periods (2001–2003 vs. 2006)

	**Published from 2001–2003**		**Published in 2006**		
			
**Reporting characteristic**	**Count**	**Percent**	**Count**	**Percent**	**P-Value**^a^
**Basic descriptive items**					

Number of study participants					
< 100	49	15.6	5	17.9	0.90
100–499	190	60.3	16	57.1	
500–999	47	14.9	4	14.3	
>= 1000	29	9.2	3	10.7	
Number of genes analyzed					
1	188	59.7	21	75.0	0.12
2	71	22.5	4	14.3	
>= 3	56	17.8	3	10.7	
Number of genetic variants analyzed					
1	114	36.2	10	35.7	0.29
2	85	27.0	4	14.3	
3	56	17.8	6	21.4	
4	21	6.6	1	3.6	
>= 5	39	12.4	7	25.0	
Number of environmental factors examined in gene-environment interactions					
1	34	70.8	1	50.0	0.26
2	10	20.8	1	50.0	
>= 3	4	8.4	0	0.0	

**Items applicable to all articles that were addressed in less than 50% of the 2001–2003 HuGE articles**					

Reported the available power of the study					
No	275	87.3	20	71.4	0.03
Yes	40	12.7	8	28.6	
Explicitly stated the use of unrelated study participants					
No	259	82.2	18	64.3	0.03
Yes	56	17.8	10	35.7	
Reported that genotyping results were validated using duplicate samples					
No	293	93.0	22	78.6	0.02
Yes	22	7.0	6	21.4	
Reported that genotyping results were validated using a different method					
No	284	90.2	24	85.7	0.32
Yes	31	9.8	4	14.3	
Reported that the evaluation of the genetic test was blind to outcomes or phenotypes					
Blind	35	11.1	3	10.7	0.62
Unclear	280	88.9	25	89.3	
Reported that the evaluation of the outcomes or phenotypes was blind to the genetic test					
Blind	12	3.8	2	7.1	0.32
Unclear	303	96.2	26	92.9	
Reported that individuals were excluded from the original group(s) of study participants					
No	278	88.2	26	92.9	0.86
Yes	37	11.8	2	7.1	
Reported that several samples could not be genotyped					
No	266	84.4	23	82.1	0.46
Yes	49	15.6	5	17.9	
Reported that unlinked genetic markers were used to identify population stratification					
No	313	99.4	28	100.0	1.00
Yes	2	0.6	0	0.0	
Reported that all genetic variants were examined for Hardy-Weinberg equilibrium					
No	164	52.1	12	42.9	0.23
Yes	151	47.9	16	57.1	

^a ^P-values were determined from two-sided Mann-Whitney U tests for the basic descriptive items and one sided Fisher's Exact Tests for the items that were reported in less than 50% of the 2001–2003 articles.

Three of the ten items that were reported in fewer than 50 percent of the articles from 2001–2003 were reported significantly more often in the 2006 articles (Table 7). Studies published in 2006 were more likely to report the available power of the study (2001–2003: 12.7 percent; 2006: 28.6 percent; p = .03), the use of unrelated study participants (2001–2003: 17.8 percent; 2006: 35.7 percent; p = .03), and the validation of genotypic results using duplicate samples (2001–2003: 7.0 percent; 2006: 21.4 percent; p = .02). Nevertheless, every item except for Hardy-Weinberg equilibrium was reported in fewer than half of all articles in the 2006 sample.

## Discussion

Many published claims of gene-disease association have not been replicated when studied in independent samples [5,6]. Suspected causes of this inconsistency include the assessment of statistical significance without accounting for the low prior probability of association, low statistical power, improper selection of participants, measurement error, confounding, and the selective reporting of results in the published literature [1,2,5,6,10-15]. Previous analyses have found that many published articles in genetic epidemiology do not provide sufficient information to evaluate these causes [16-18]. However, the results of these analyses were limited to a specific phenotypic outcome (e.g., sepsis) [16,17] or are outdated [18]. Our analysis provides an updated review of reporting on these key elements in two representative samples of HuGE articles.

Representative of the literature in this field, most of the studies in our samples were small: only about 10 percent of the studies reported sample sizes that exceeded 1,000. Several meta-analyses have found significant differences between the results of small and large genetic association studies [6,19]. Growing evidence suggests that individual genetic variants impart only a modest effect on the risk of developing complex, multifactorial diseases [20-22]. Thus, enrolling many thousands or even tens of thousands of individuals may be required to achieve the necessary power to identify and validate true genetic associations [13,20,23,24].

Our ability to assess the potential for selection biases was severely limited in many of the studies we examined. Although most studies provided some qualitative descriptions of the study participants (such as origin and enrollment criteria), reporting was sporadic for even simple descriptors, such as age and sex. Potentially important details, such as the number of exclusions or the number of samples that could not be genotyped, were often omitted.

Misclassification can severely limit study power and bias the results [14,23,25-27]. We determined about a tenth of studies may have misclassified their phenotypic outcomes and three-fourths of studies may have misclassified their environmental factors. A small proportion of studies reported measures such as genotyping replicate samples and blinding the research staff [27,28] to help ensure that the genetic data were not misclassified. Although the practice of detecting genotyping errors through tests of Hardy-Weinberg Equilibrium is still being debated [27,29-31], approximately half of studies reported HWE test results.

Population stratification may occur when study participants are selected from subpopulations with a different prevalence of the phenotypes and genotypes [21,30,32,33]. Although the extent to which population stratification contributes to spurious findings remains debatable [34-36], most of the articles in our sample provided descriptions for the ethnic origin of the study participants, and almost all case-control studies indicated that the cases and controls were drawn from the same population. A few studies reported the use of unlinked markers to provide evidence that population stratification was not an issue in the analysis. Genome-wide association studies provide considerable genetic data to examine and correct for population stratification (e.g. by principal component analysis) [37-39].

As a result of the selective reporting of significant results from multiple analyses and publication bias, the extent of type I error in the published literature may be great [11,14,21,23,26,28,30,40,41]. Although most studies reported results for only a few polymorphisms and environmental factors, it is difficult to determine the number actually tested; only a minority of articles reported using corrections for multiple comparisons, even for the reported associations. Reporting justifications for specific genetic comparisons could suggest that studies were founded on an a priori hypothesis and were not the result of selective reporting. However, less than one-half of the studies that assessed dominant or recessive genetic models provided justifications for the use of these specific comparisons and not others. Among articles that described gene-gene or gene-environment interactions, a substantial proportion reported statistically significant results. However, many of these may be spurious, given the limited power of most studies to identify true associations, let alone interaction effects [20,32]. The high frequency of "positive" results in our study sample could reflect a combination of multiple testing, selective reporting, and publication bias [14].

The use of common reporting standards could increase the transparency of research methodology, thus helping to identify selective reporting and sources of bias and confounding while allowing for a more complete synthesis of data across consortia or in meta-analyses [1,10,42]. The results of our study were presented at a HuGENet sponsored workshop, Strengthening the Reporting of Genetic Associations (STREGA) [43]. The workshop concluded with an agreement to develop an extension of the STROBE (STrengthening the Reporting of OBservational studies in Epidemiology) statement [44] to address some of the specific challenges (e.g., generation of genetic data, population stratification, haplotype inference, HWE, and multiple testing) posed by reporting results of genetic epidemiology studies. STREGA is currently finalized for publication, as of writing this article.

By publishing supplementary information online, journals increasingly provide authors the opportunity to present their study methods and results in greater detail than is permitted in print [45,46]. Recently, an increase in such supplements–often used to report additional methods, tables, and figures–was documented for a number of high impact journals [45]. However, authors and journals need to ensure that this information remains available to readers and is not lost in broken links [46].

## Conclusion

In summary, our results provide evidence that many details needed to assess the validity of study findings are not consistently reported in human genome epidemiology studies, though some improvement has been seen recently. The use of standard reporting guidelines and online supplements could help readers to better judge the scientific evidence. As large-scale genotyping platforms are rapidly introduced in human genome epidemiology, the importance of transparent reporting of the background, epidemiological methods, and population characteristics cannot be understated given the challenge of assessing, interpreting, and discussing ever-greater amounts of data.

## Competing interests

The authors declare that they have no competing interests.

## Authors' contributions

AY assisted in data collection, performed the analysis, and drafted the manuscript. EE, FKK, and NAP assisted in data collection and the revision of the manuscript. MC identified and indexed relevant articles for the HuGE Published Literature database. MCW and BKL created the standardized abstraction form and assisted in the sampling process and data collection. WY curates the HuGE Published Literature and assisted in the sampling process. MG, JPAI, and MJK designed the study, oversaw the project, and revised the manuscript. All authors read and approved the final manuscript.

## Note

The findings and conclusions in this report are those of the authors and do not necessarily represent the views of the Centers for Disease Control and Prevention.

## Pre-publication history

The pre-publication history for this paper can be accessed here:


